# Scientific breakdown for physiological blood flow inside a tube with multi-thrombosis

**DOI:** 10.1038/s41598-021-86051-2

**Published:** 2021-03-24

**Authors:** Salman Akhtar, L. B. McCash, Sohail Nadeem, Anber Saleem

**Affiliations:** 1 Department of Mathematics, Quaid-i-Azam University 45320, Islamabad, 44000 Pakistan; 2 School of Mathematics & Acturial Science, University of Leicester, Leicester, LE1 7RH UK; 3 Department of Anatomy, School of Dentistry, SZABMU, Islamabad, Pakistan

**Keywords:** Computational biophysics, Biophysics, Mathematics and computing

## Abstract

The blood flow inside a tube with multi-thromboses is mathematically investigated. The existence of these multiple thromboses restricts the blood flow in this tube and the flow is revamped by using a catheter. This non-Newtonian blood flow problem is modeled for Jeffrey fluid. The energy equation includes a notable effect of viscous dissipation. We have calculated an exact solution for the developed mathematical governing equations. These mathematical equations are solved directly by using Mathematica software. The graphical outcomes are added to discuss the results in detail. The multiple thromboses with increasing heights are evident in streamline graphs. The sinusoidally advancing wave revealed in the wall shear stress graphs consists of crest and trough with varying amplitude. The existence of multi-thrombosis in this tube is the reason for this distinct amplitude of crest and trough. Further, the viscous dissipation effects come out as a core reason for heat production instead of molecular conduction.

## Introduction

The phenomenon that explains the transport of biological fluid inside a tube with sinusoidally moving walls is known as Peristalsis. Barton^1^ had studied the peristaltic flow with the assumption of long peristaltic wavelength. The different peristaltic flow properties and assumptions like creeping movement were discussed in the study of Pozrikidis^2^. The peristalsis mechanism can also happen within a vessel having a short length, as the diameter of such vessels alters systematically due to vasomotion^3^. The peristalsis mechanism is a vast study area of interest, as it has major applications and uses in engineering and biomedical problems. This phenomenon is mainly used in many devices that work as blood pumps, transport of sludge as well as food and different biological liquids^4^. The non-Newtonian fluid models are used by many of the researchers to study peristaltic blood flow problems. Mekheimer^5^ had utilized the non-Newtonian study model to examine the unsteady, two-dimensional, peristaltic flow of blood. Further, the theoretical work that provides the flow across channels with sinusoidal advancing walls, using a non-Newtonian study model is given^6,7^.

In blood vessels, some blood particles that get attached to the wall of a vessel, when these particles detach from the wall to again join the stream of blood then such particles may form a blood clot. The flow is refined in such conditions with catheter application in such tubes. Mekheimer et al.^8^ had investigated the blood flow inside a catheterized cylindrical geometry with thrombus and peristaltic effects. The mathematical investigation of peristaltic flow with clot applications through an annular section was conveyed by Nadeem et al.^9^. Bhatti^10^ had utilized a non-Newtonian study model to interpret the flow across an annular region with thrombus and peristaltic applications. The investigation of the heat transfer phenomenon for annular blood flow problems together with combined applications of peristalsis and thrombus at the center of the tube also has remarkable importance due to its applications. Akbar^11^ had conveyed a mathematical investigation for the heat transport mechanism of peristaltic flow through an annular section using a non-Newtonian study model. The flow through an annular region having sinusoidally advancing exterior walls with heat transport effects was mathematically examined by Vajravelu^12^. The heat transport mechanism for concentric cylinders with the outer cylinder having a sinusoidal wave, using Jeffrey model of non-Newtonian fluid flow was investigated by Vasudev et al.^13^. Some more recent studies that provide the analysis of heat transfer and blood flow are cited as^14–21^.

We have thoroughly investigated the already available research articles and this observation clearly shows that the peristaltic flow of blood within a channel having multi-thrombosis is not mathematically investigated by anyone. To cover this gap in the literature, the peristaltic blood flow inside a tube with multi-thrombosis is mathematically investigated for the first time. The existence of these multiple thromboses restricts the blood flow across the tube and the flow is revamped by using a catheter. This non-Newtonian blood flow problem is modeled for Jeffrey fluid. We have gained an exact solution for the developed mathematical governing equations. The graphical results are added to discuss these exact results in detail.

## Mathematical model

**Figure 1:**
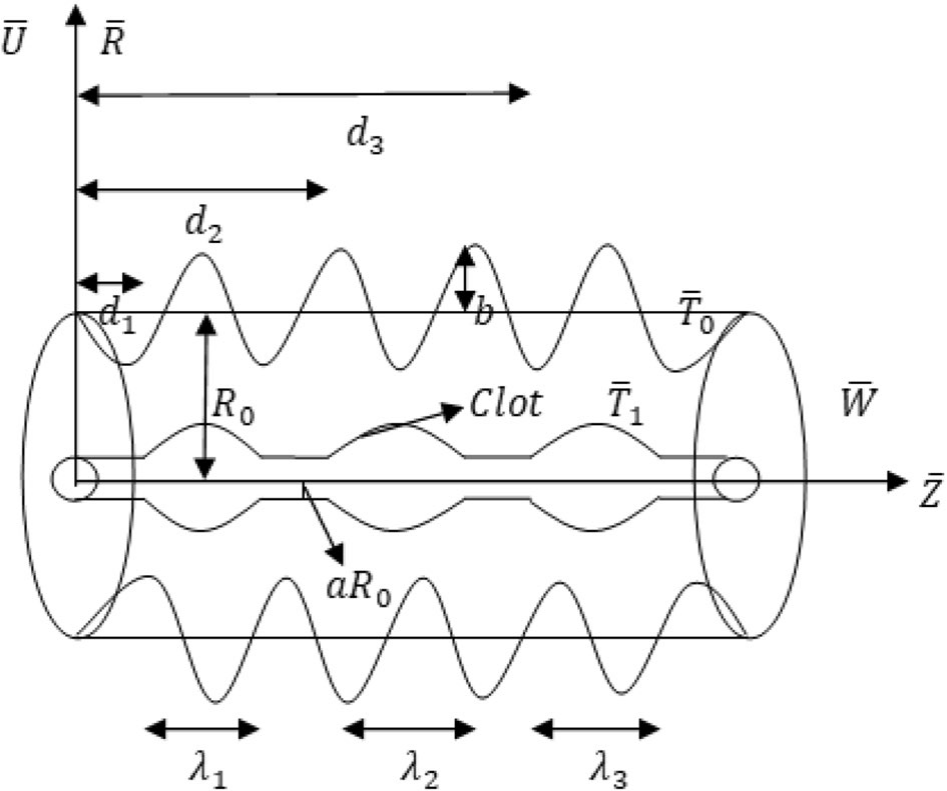
Geometry of the problem.

The peristaltic blood flow inside a geometry with multi-thrombosis is mathematically investigated. The presence of these multiple clots reduces the blood flow through the tube and the flow of blood is revamped by using a catheter (See Fig. 1).

The tube’s outer surface $\overline{\eta }$ (z) with a traveling sinusoidal wave and the inner surface $\overline{\epsilon }(z)$ having multiple clots is provided with their dimensional mathematical expressions

1 $$ \overline{\eta }\left( z \right) = R_{0} + b\;Sin\left( {\frac{2\pi }{\lambda }\left( {\overline{Z} - c\overline{t}} \right)} \right), $$

2 $$ \overline{{\epsilon}} \left( z \right) = \left\{ {\begin{array}{*{20}l} {R_{0} \left[ {a + f_{1} \left( {\overline{z}} \right)} \right],} \hfill & {d_{l} \le \overline{z} \le d_{l} + \lambda_{l} } \hfill \\ {R_{0} a} \hfill & {otherwise} \hfill \\ \end{array} } \right., $$

here ${f}_{1}(\overline{z})$ defines the geometry of multi-thrombosis.

The dimensional form of formulated equations is

3 $$\frac{\partial \overline{U}}{\partial \overline{R}}+\frac{\overline{U}}{\overline{R}}+\frac{\partial \overline{W}}{\partial \overline{Z}}=0,$$

4 $$\rho \left(\frac{\partial \overline{U}}{\partial \overline{t}}+\overline{U}\frac{\partial \overline{U}}{\partial \overline{R}}+\overline{W}\frac{\partial \overline{U}}{\partial \overline{Z}}\right)=-\frac{\partial \overline{P}}{\partial \overline{R}}+\frac{1}{\overline{R}}\frac{\partial }{\partial \overline{R}}\left(\overline{R}{\overline{S}}_{\overline{R}\overline{R}}\right)+\frac{\partial }{\partial \overline{Z}}\left({\overline{S}}_{\overline{R}\overline{Z}}\right),$$

5 $$\rho \left(\frac{\partial \overline{W}}{\partial \overline{t}}+\overline{U}\frac{\partial \overline{W}}{\partial \overline{R}}+\overline{W}\frac{\partial \overline{W}}{\partial \overline{Z}}\right)=-\frac{\partial \overline{P}}{\partial \overline{Z}}+\frac{1}{\overline{R}}\frac{\partial }{\partial \overline{R}}\left(\overline{R}{\overline{S}}_{\overline{R}\overline{Z}}\right)+\frac{\partial }{\partial \overline{Z}}\left({\overline{S}}_{\overline{Z}\overline{Z}}\right),$$

6 $$\rho {C}_{p}\left(\frac{\partial \overline{T}}{\partial \overline{t}}+\overline{U}\frac{\partial \overline{T}}{\partial \overline{R}}+\overline{W}\frac{\partial \overline{T}}{\partial \overline{Z}}\right)={\overline{S}}_{\overline{R}\overline{R}}\frac{\partial \overline{U}}{\partial \overline{R}}+{\overline{S}}_{\overline{R}\overline{Z}}\frac{\partial \overline{W}}{\partial \overline{R}}+{\overline{S}}_{\overline{Z}\overline{R}}\frac{\partial \overline{U}}{\partial \overline{Z}}+{\overline{S}}_{\overline{Z}\overline{Z}}\frac{\partial \overline{W}}{\partial \overline{Z}}+k\left(\frac{{\partial }^{2}\overline{T}}{\partial {\overline{R}}^{2}}+\frac{1}{\overline{R}}\frac{\partial \overline{T}}{\partial \overline{R}}+\frac{{\partial }^{2}\overline{T}}{\partial {\overline{Z}}^{2}}\right).$$

The Jeffrey fluid tensor for extra stresses is taken as^22^

7 $$\overline{S}=\frac{\mu }{1+{\lambda }_{1}}\left(\dot{\gamma }+{\lambda }_{2}\ddot{\gamma }\right),$$

The fixed and moving frame is correlated by the following equations

8 $$ \overline{r} = \overline{R},\;\overline{z} = \overline{Z} - c\overline{t},\;\overline{u} = \overline{U},\;\overline{w} = \overline{W} - c,\;\overline{p}\left( {\overline{z},\overline{r}} \right) = \overline{P}\left( {\overline{Z},\overline{R},\overline{t}} \right), $$

The used non-dimensional variables are provided as

9 $$r=\frac{\overline{r}}{{R}_{0}}, z=\frac{\overline{z}}{{\lambda }_{l}}, u=\frac{{\lambda }_{l}\overline{u}}{{R}_{0}c}, w=\frac{\overline{w}}{c}, t=\frac{c\overline{t}}{{\lambda }_{l}}, p=\frac{{R}_{0}^{2}\overline{p}}{c{\lambda }_{l}{\mu }_{f}}, \theta =\frac{\overline{T}-{\overline{T}}_{0}}{{\overline{T}}_{1}-{\overline{T}}_{0}}, \epsilon \left(z\right)=\frac{\overline{\epsilon }\left(z\right)}{{R}_{0}}, \eta \left(z\right)=\frac{\overline{\eta }\left(z\right)}{{R}_{0}}, \phi =\frac{b}{{R}_{0}}, {B}_{r}=\frac{{c}^{2}{\mu }_{f}}{\left({\overline{T}}_{1}-{\overline{T}}_{0}\right){k}_{f}},S=\frac{{R}_{0}}{\mu c}\overline{S},{h}_{l}=\frac{{d}_{l}}{{\lambda }_{l}},$$

Equations (4–6) provides the following dimensionless equation after the application of Eqs. (8) and (9)

10 $$\frac{\partial p}{\partial r}=0,$$

11 $$\frac{dp}{dz}=\frac{1}{1+{\lambda }_{1}}\left(\frac{{\partial }^{2}w}{\partial {r}^{2}}+\frac{1}{r}\frac{\partial w}{\partial r}\right),$$

12 $$\frac{{\partial }^{2}\theta }{\partial {r}^{2}}+\frac{1}{r}\frac{\partial \theta }{\partial r}+\left(\frac{1}{1+{\lambda }_{1}}\right){B}_{r}{\left(\frac{\partial w}{\partial r}\right)}^{2}=0,$$

The appropriate non-dimensional boundary conditions are

13 $$  w = - 1\;at\;r = {\epsilon} \left( z \right)\;and\;w = - 1\;at\;r = \eta \left( z \right), $$

14 $$\theta = 1\;at\;r = {\epsilon} \left( z \right)\;and\;\theta = 0\;at\;r = \eta \left( z \right), $$

In boundary condition (13), we have $w = - 1\;at\;r = {\epsilon}\left( z \right)\;and\;w = - 1\;at\;r = \eta \left( z \right).$ The velocity “w” takes the value (minus one) in the dimensionless form. Therefore, in the graphical results of velocity we see negative values that exactly approach to minus one. Further, In the dimensional form we have set $\overline{W}=0$ and then by using the transformation $\overline{w}=\overline{W}-c$ given in Eq. (8), we get $\overline{w}=-c$ and then by using $w=\frac{\overline{w}}{c}$ given in Eq. (9), we finally get $w=-1$.

The exterior surface $\eta \left(z\right)$ and the interior surface $\epsilon (z)$ with their dimensionless mathematical expressions are provided. The expression for ${f}_{1}(\overline{z})$ is chosen as^23^

15 $$ \eta \left( z \right) = 1 + \phi \;Sin\left( {2\pi z} \right), $$

16 $$  {\epsilon}\left( z \right) = \left\{ {\begin{array}{*{20}l} {a + \sigma_{l} e^{{ - \pi^{2} \left( {z - z_{{d_{l} }} - 0.5} \right)^{2} }} ,} \hfill & {h_{l} \le z \le h_{l} + 1} \hfill \\ a \hfill & {otherwise} \hfill \\ \end{array} } \right., $$

## Exact solution

The velocity profile $w\left(r,z\right)$ is exactly solved to get

17 $$w\left(r,z\right)=\frac{1}{4(log\left(\epsilon \right)-log(\eta ))}\left[4\left(log\left(\eta \right)-log\left(\epsilon \right)\right)+\frac{dp}{dz}(1+{\lambda }_{1})\left\{\left({\eta }^{2}-{\epsilon }^{2}\right)log\left(r\right)+\left({r}^{2}-{\eta }^{2}\right)log\left(\epsilon \right)+\left({\epsilon }^{2}-{r}^{2}\right)log(\eta )\right\}\right],$$

where log represents the logarithmic function.

The rate for volume flow between these two walls is

18 $$Q=2\pi \underset{\epsilon }{\overset{\eta }{\int }}rwdr,$$

Finally, by using volume flow rate calculations, we get pressure gradient as

19 $$\frac{dp}{dz}=\frac{8\left(Q+\pi ({-\epsilon }^{2}+{\eta }^{2})\right)(log\left(\epsilon \right)-log(\eta ))}{\pi \left({\epsilon }^{2}-{\eta }^{2}\right)\left(1+{\lambda }_{1}\right)\left\{-{\epsilon }^{2}+{\eta }^{2}+\left({\epsilon }^{2}+{\eta }^{2}\right)(log\left(\epsilon \right)-log(\eta ))\right\}},$$

The result for ${\tau }_{w}$ is calculated as follows

20 $${\tau }_{w}=-{\left.\frac{\partial w}{\partial r}\right|}_{r=\eta }=-\frac{\frac{dp}{dz}(1+{\lambda }_{1})\left(\frac{-{\epsilon }^{2}+{\eta }^{2}}{\eta }+2\eta (log\left(\epsilon \right)-log(\eta ))\right)}{4(log\left(\epsilon \right)-log(\eta ))},$$

The temperature solution is also solved exactly and the expression is given by

21 $$ \begin{aligned}\theta \left(r,z\right)&=\frac{1}{64{(log\left(\epsilon \right)-log(\eta ))}^{2}}\left[-2{B}_{r}{\left(\frac{dp}{dz}\right)}^{2}{\left({\epsilon }^{2}-{\eta }^{2}\right)}^{2}\left(1+{\lambda }_{1}\right){\left(log\left(r\right)\right)}^{2}\right.\\&\quad\left.-{B}_{r}{\left(\frac{dp}{dz}\right)}^{2}\left({r}^{2}-{\eta }^{2}\right)\left(1+{\lambda }_{1}\right)log\left(\epsilon \right)\left(-4\left({\epsilon }^{2}-{\eta }^{2}\right)+\left({r}^{2}+{\eta }^{2}\right)log\left(\epsilon \right)\right)\right.\\&\quad \left.+\left\{-4{B}_{r}{\left(\frac{dp}{dz}\right)}^{2}\left({r}^{2}-{\epsilon }^{2}\right)\left({\epsilon }^{2}-{\eta }^{2}\right)\left(1+{\lambda }_{1}\right)\right.\right.\\&\quad\left.\left.+\left(-64+{B}_{r}{\left(\frac{dp}{dz}\right)}^{2}\left(2{r}^{4}-3{\epsilon }^{4}+4{\epsilon }^{2}{\eta }^{2}-3{\eta }^{4}\right)\left(1+{\lambda }_{1}\right)\right)log\left(\epsilon \right)\right\}log\left(\eta \right)\right.\\&\quad\left.+\left(64-{B}_{r}{\left(\frac{dp}{dz}\right)}^{2}\left({r}^{4}-{\epsilon }^{4}\right)(1+{\lambda }_{1})\right){(log(\eta ))}^{2}\right.\\&\quad \left.+log(r)\left\{-4{B}_{r}{\left(\frac{dp}{dz}\right)}^{2}{\left({\epsilon }^{2}-{\eta }^{2}\right)}^{2}\left(1+{\lambda }_{1}\right)+\left(64+{B}_{r}{\left(\frac{dp}{dz}\right)}^{2}\left(3{\epsilon }^{4}-4{\epsilon }^{2}{\eta }^{2}+{\eta }^{4}\right)\left(1+{\lambda }_{1}\right)\right)log\left(\epsilon \right)\right.\right.\\&\quad \left.\left.+\left(-64+{B}_{r}{\left(\frac{dp}{dz}\right)}^{2}\left({\epsilon }^{4}-4{\epsilon }^{2}{\eta }^{2}+3{\eta }^{4}\right)\left(1+{\lambda }_{1}\right)\right)log(\eta )\right\}\right],\end{aligned} $$

## Results and discussion

**Figure 2:**
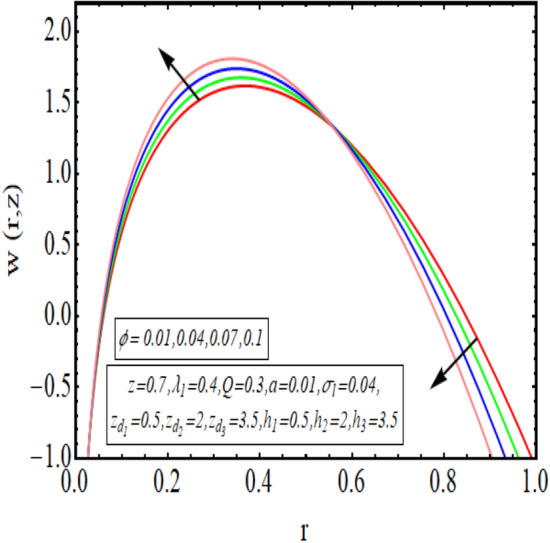
Velocity for $$\phi $$.

**Figure 3:**
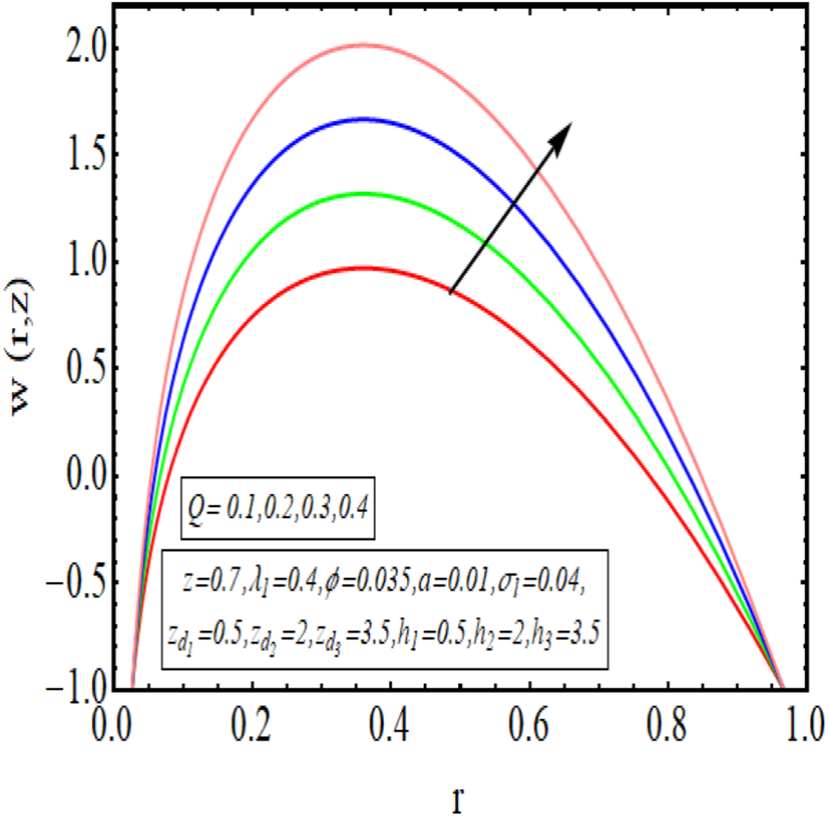
Velocity for $$Q$$.

**Figure 4:**
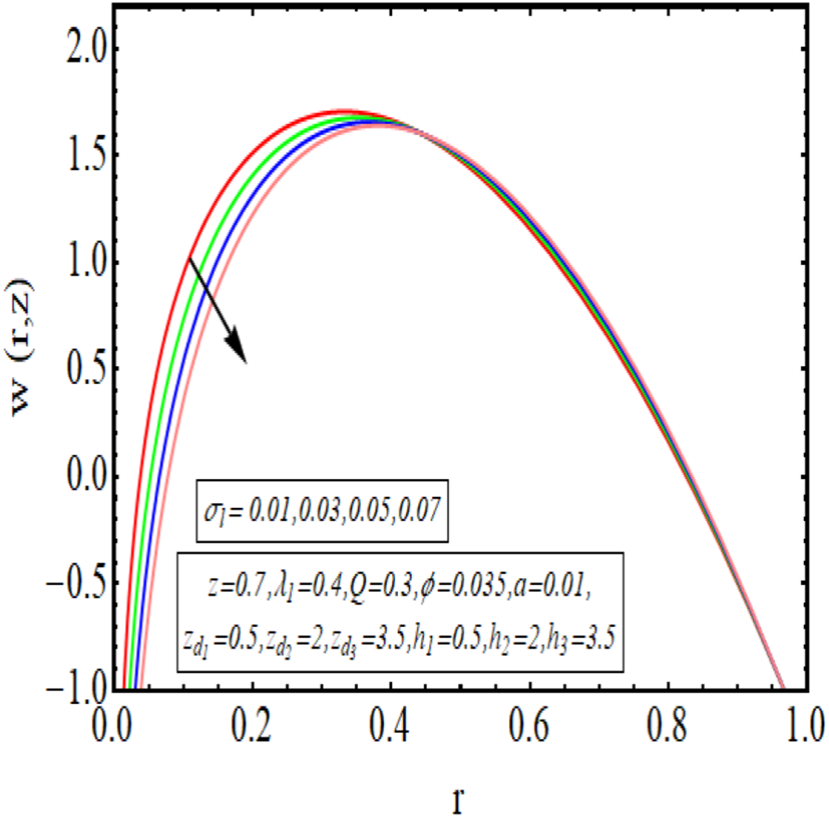
Velocity for $${\sigma }_{l}$$.

**Figure 5:**
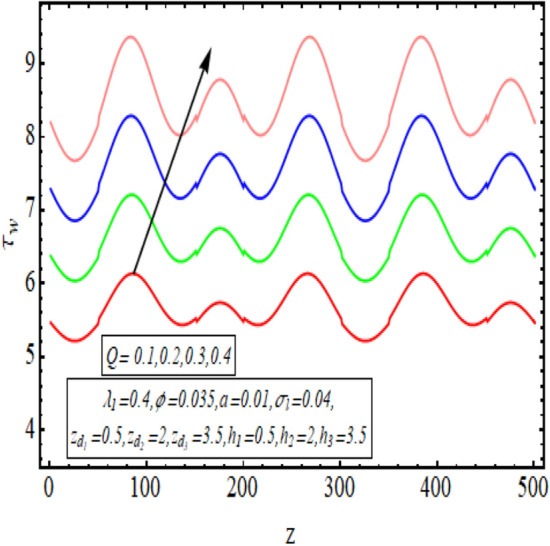
$${\tau }_{w}$$ for $$Q$$.

**Figure 6:**
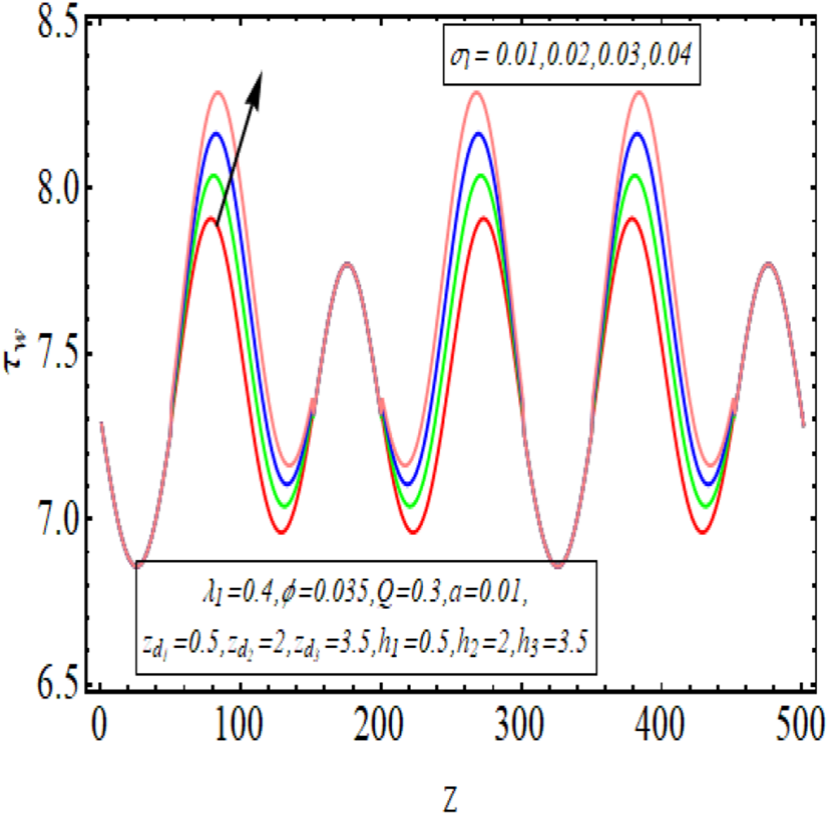
$${\tau }_{w}$$ for $${\sigma }_{l}$$.

**Figure 7:**
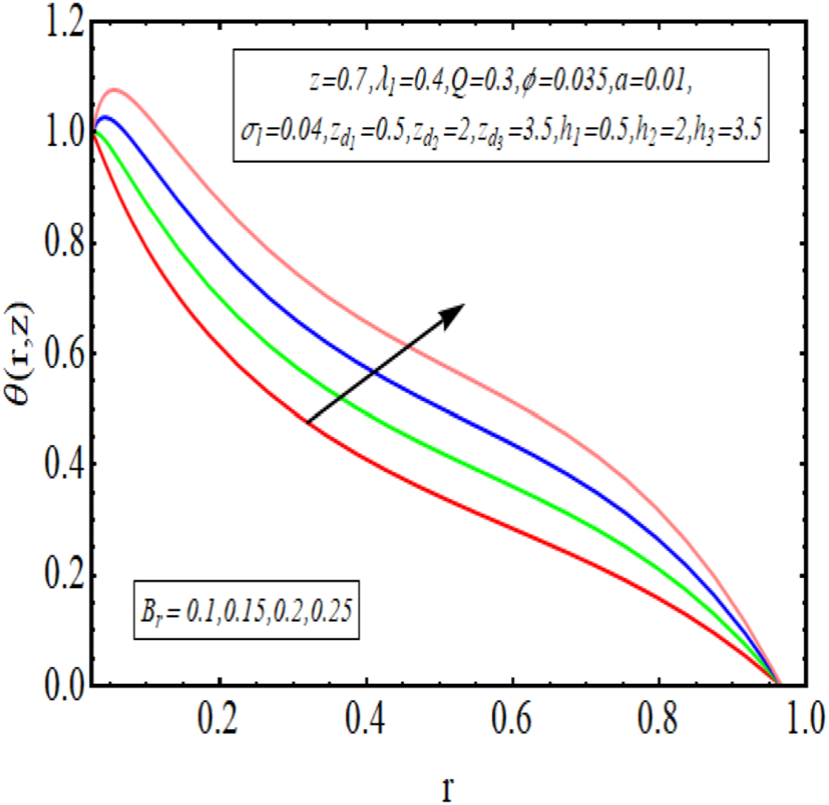
Temperature profile for ${B}_{r}$.

**Figure 8:**
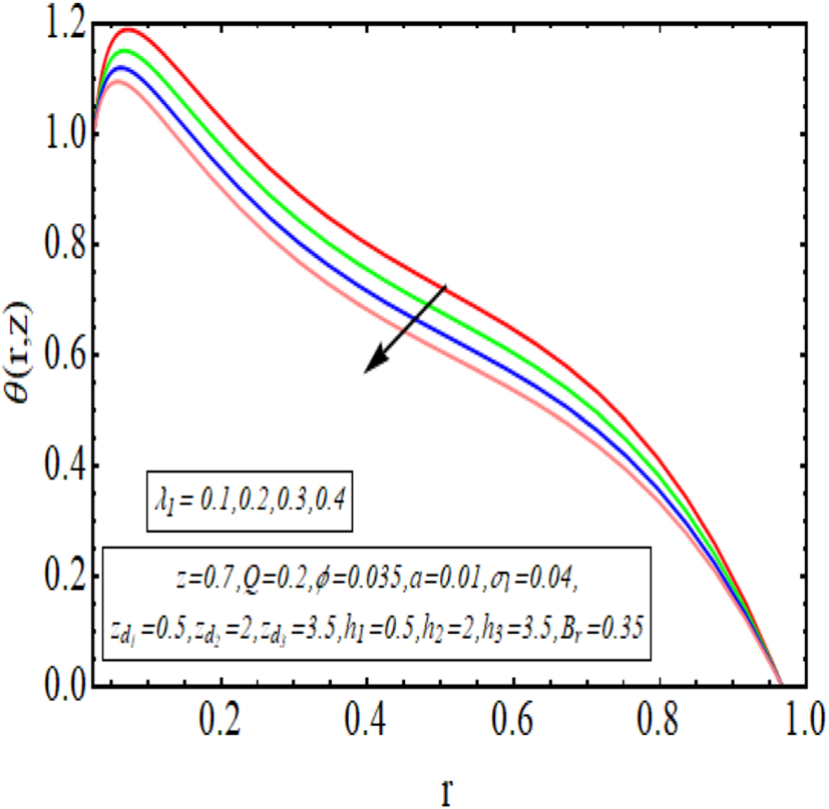
Temperature profile for ${\lambda }_{1}$.

**Figure 9:**
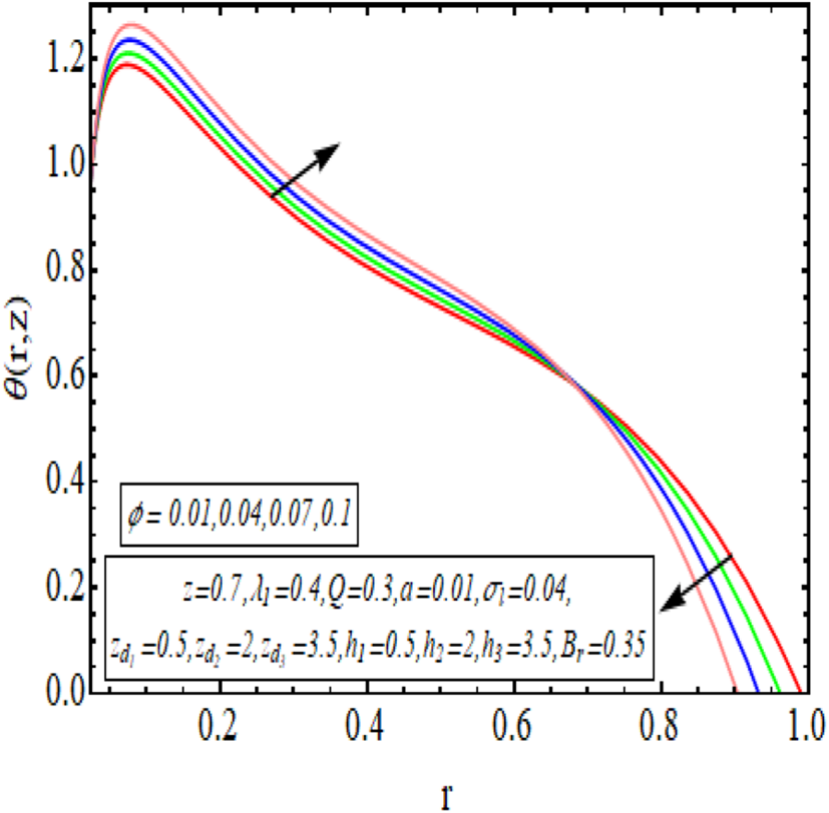
Temperature profile for $\phi$.

**Figure 10:**
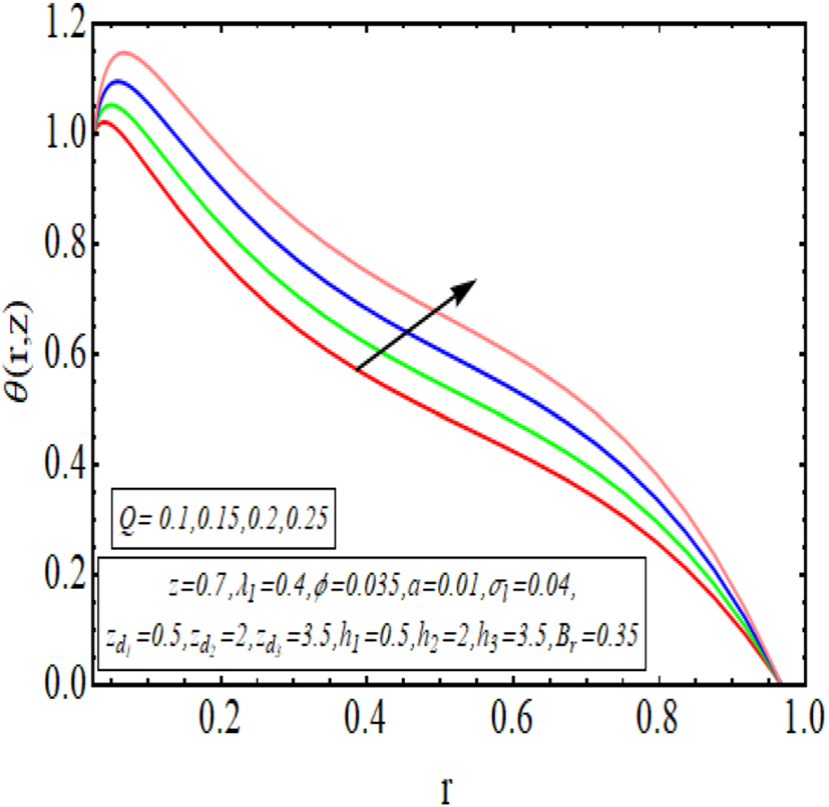
Temperature profile for $Q$.

**Figure 11:**
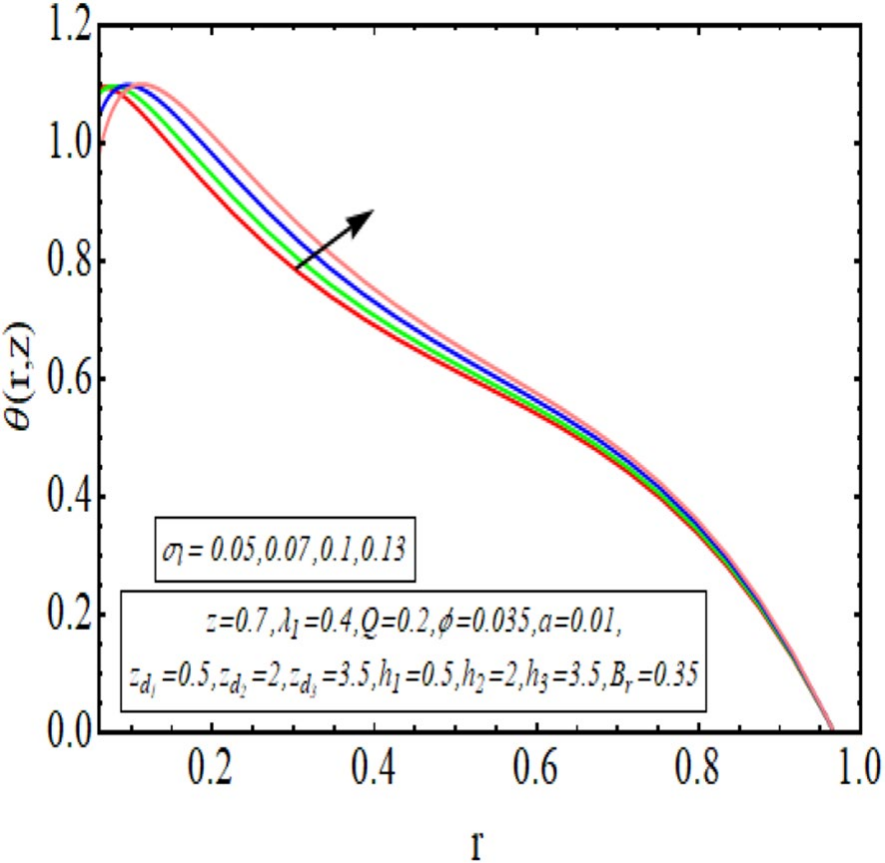
Temperature profile for ${\sigma }_{l}$.

**Figure 12:**
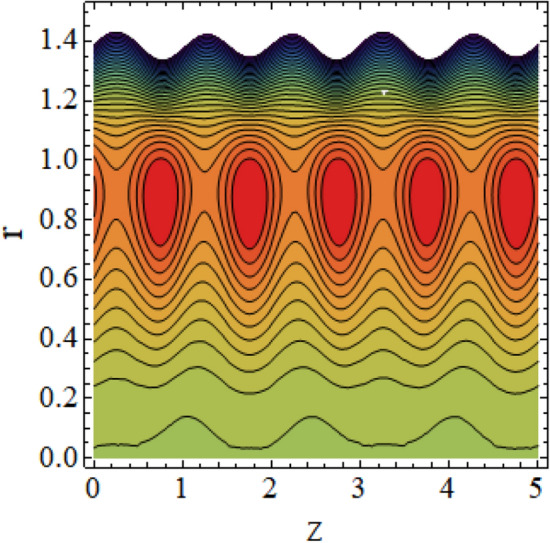
Streamlines for $$Q = 0.5\;{\text{with}}\;\lambda_{1} = 0.4,\;\phi = 0.037,\;a = 0.01,\;z_{{d_{1} }} = 0.5,\;z_{{d_{2} }} = 2,\;z_{{d_{3} }} = 3.5,\;h_{1} = 0.5,\;h_{2} = 2,\;h_{3} = 3.5.$$

**Figure 13:**
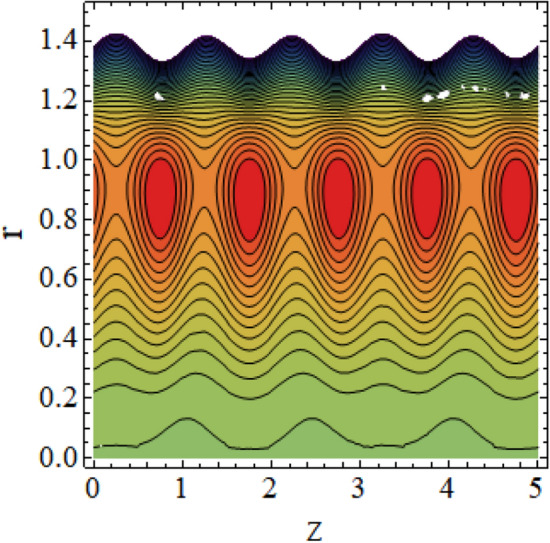
Streamlines for $$Q = 0.6\;{\text{with}}\;\lambda_{1} = 0.4,\;\phi = 0.037,\;a = 0.01,\;z_{{d_{1} }} = 0.5,\;z_{{d_{2} }} = 2,\;z_{{d_{3} }} = 3.5,\;h_{1} = 0.5,\;h_{2} = 2,\;h_{3} = 3.5.$$

**Figure 14:**
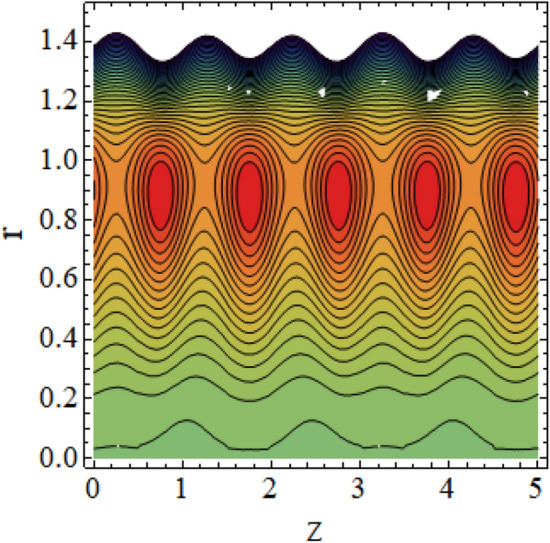
Streamlines for $Q = 0.7$ with $\lambda_{1} = 0.4,\;\phi = 0.037,\;a = 0.01,\;z_{{d_{1} }} = 0.5,\;z_{{d_{2} }} = 2,\;z_{{d_{3} }} = 3.5,\;h_{1} = 0.5,\;h_{2} = 2,\;h_{3} = 3.5.$

**Figure 15:**
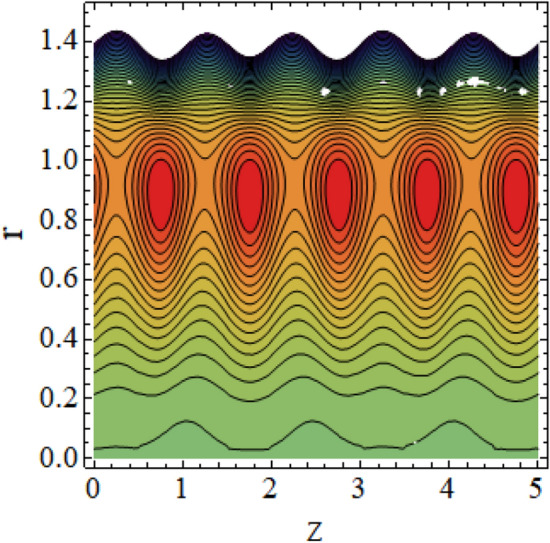
Streamlines for $$Q = 0.75\;{\text{with}}\;\lambda_{1} = 0.4,\;\phi = 0.037,\;a = 0.01,\;z_{{d_{1} }} = 0.5,\;z_{{d_{2} }} = 2,\;z_{{d_{3} }} = 3.5,\;h_{1} = 0.5,\;h_{2} = 2,\;h_{3} = 3.5.$$

**Figure 16:**
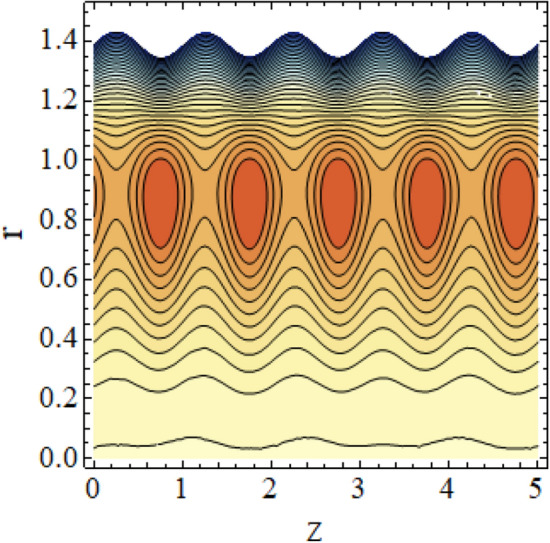
Streamlines for $\sigma_{l} = 0.01$ with $\lambda_{1} = 0.4,\;\phi = 0.037,\;a = 0.01,\;z_{{d_{1} }} = 0.5,\;z_{{d_{2} }} = 2,\;z_{{d_{3} }} = 3.5,\;h_{1} = 0.5,\;h_{2} = 2,\;h_{3} = 3.5,\;Q = 0.01.$

**Figure 17:**
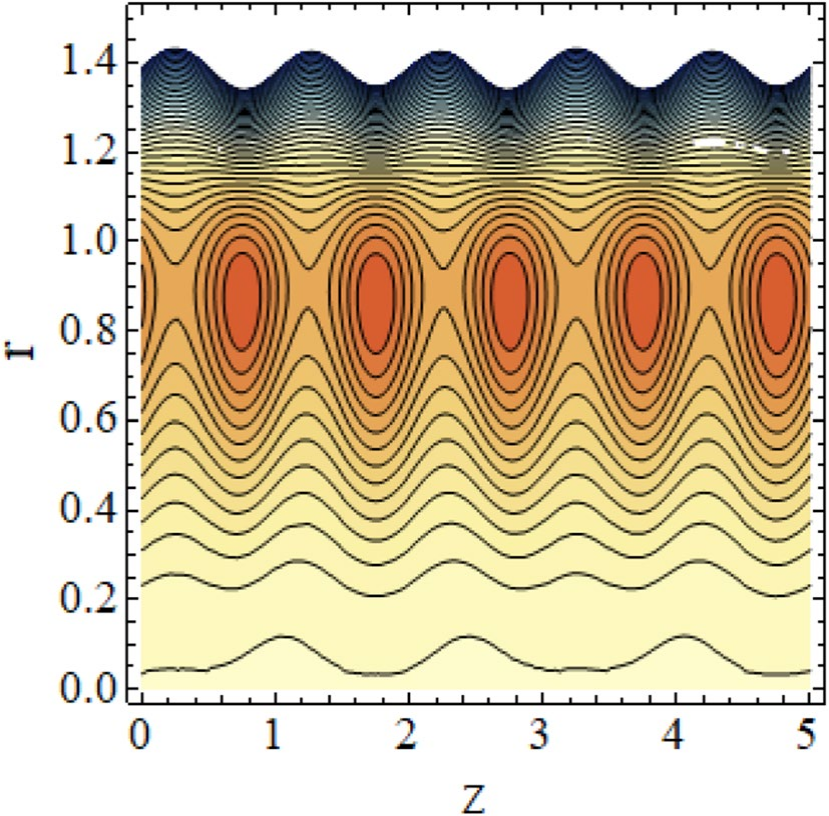
Streamlines for $\sigma_{l} = 0.03$ with $\lambda_{1} = 0.4,\;\phi = 0.037,\;a = 0.01,\;z_{{d_{1} }} = 0.5,\;z_{{d_{2} }} = 2,\;z_{{d_{3} }} = 3.5,\;h_{1} = 0.5,\;h_{2} = 2,\;h_{3} = 3.5,\;Q = 0.01.$

**Figure 18:**
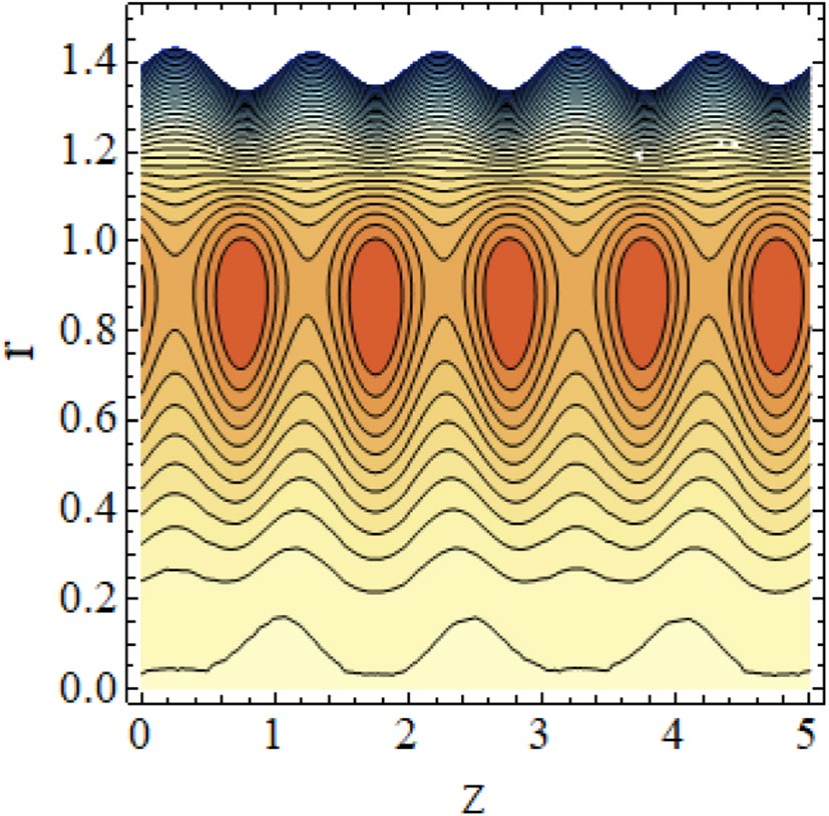
Streamlines for $\sigma_{l} = 0.05$ with $\lambda_{1} = 0.4,\;\phi = 0.037,\;a = 0.01,\;z_{{d_{1} }} = 0.5,\;z_{{d_{2} }} = 2,\;z_{{d_{3} }} = 3.5,\;h_{1} = 0.5,\;h_{2} = 2,\;h_{3} = 3.5,\;Q = 0.01.$

**Figure 19:**
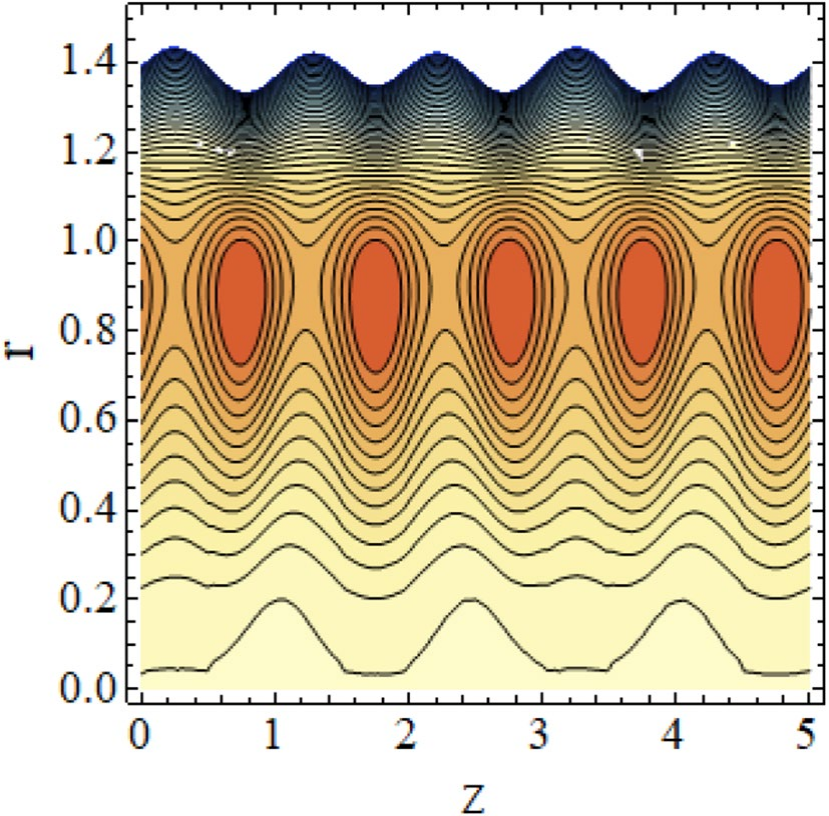
Streamlines for $\sigma_{l} = 0.07$ with $\lambda_{1} = 0.4,\;\phi = 0.037,\;a = 0.01,\;z_{{d_{1} }} = 0.5,\;z_{{d_{2} }} = 2,\;z_{{d_{3} }} = 3.5,\;h_{1} = 0.5,\;h_{2} = 2,\;h_{3} = 3.5,\;Q = 0.01.$

The interpreted results are discussed in detail with graphical outcomes. The velocity profile graphs are plotted and provided in Figs. 2, 3 and 4. Figure 2 represents that the velocity profile gains a higher value almost near the central region of two walls, but it shows declining behavior near the peristaltic surface with increasing $\phi$. The peristaltic transport is increased automatically with increasing the amplitude of the peristaltic wave, as this flow mainly depends on the amplitude of the peristaltic wave. Thus the velocity near the central region increases. There is an increment in the value of velocity for increasing the value of $Q$, given in Fig. 3. The velocity should gain magnitude with incrementing $Q$ as it assists the flow. Figure 4 reveals that velocity declines with a multi-thrombus wall but remains constant at the peristaltic wall for increasing values of ${\sigma }_{l}$. Figures 5 and 6 are provided to discuss the shear stress ${\tau }_{w}$ that is plotted against the axial coordinate. Figure 5 reveals that ${\tau }_{w}$ gains magnitude with an increasing value of $Q$. It is observed that the sinusoidal wave presented in this graph consists of different amplitudes crest and trough. The existence of multi-thrombosis in this tube is the reason for this distinct amplitude of crest and trough. The crest with greater amplitude depicts the position of multi-thromboses while the once with low amplitude reveal the location having no thrombus. The locations $50\le z\le 150$, $200\le z\le 300$, and $350\le z\le 450$ show the position of multi-thromboses. In Fig. 6, ${\tau }_{w}$ is plotted for an increasing value of ${\sigma }_{l}$. As the values of ${\sigma }_{l}$ increases, the value of ${\tau }_{w}$ gains magnitude exactly at positions of multi-thromboses. Thus, it is also evident from this graph that the positions of multi-thrombosis are the segments $50\le z\le 150$, $200\le z\le 300$, and $350\le z\le 450$. The graphical outcomes of temperature profile for distinct parameters are displayed in Figs. 7, 8, 9, 10 and 11. Figure 7 displays that the temperature shows increasing behavior with increasing value of ${B}_{r}$. Thus, viscous dissipation is the core reason for heat production instead of molecular conduction. Figure 8 shows that there is a decline in temperature with incrementing the value of ${\lambda }_{1}$. The temperature attains higher values with the multi-thrombus end but declines with the wavy end for enhancing the value of $\phi$, represented in Fig. 9. Figure 10 displays that temperature attains increasing values with enhancing $Q$. There is an increment in the temperature for increasing the value of ${\sigma }_{l}$, displayed in Fig. 11. Streamline graphical outcomes are plotted for increasing the value of $Q$, as given in Figs. 12, 13, 14 and 15. These graphs convey that the trapping decreases in size but increases in the count with increasing $Q$. A clear picture of the sinusoidal wave is seen at one end and multi-thrombosis at another end. These streamline graphical results (Figs. 12, 13, 14 and 15) are plotted for fixed height of multiple thrombosis and the fixed height is evident in the graphs. The next given graphical results (Figs. 16, 17, 18 and 19) are plotted for varying heights of multiple thrombosis and the variation in the height of these multiple thrombosis is noted in these graphs. In this way, we have also covered the present topic for different heights of multiple thrombosis. Figures 16, 17, 18 and 19 displays a streamlined graph for increasing values of ${\sigma }_{l}$. It is interesting to note the increase in height of multi-thrombosis in these streamlines graphs.

## Conclusions

The peristaltic blood flow inside a cylindrical geometry with multi-thrombus is mathematically investigated. The presence of these multiple clots reduces the blood flow across the tube and the flow is revamped by catheter utilization. The important outcome results are

- the velocity profile gain magnitude almost near the central region of both walls but it shows declining behavior near the peristaltic surface with increasing $\phi$.
- The velocity profile declines with the multi-thrombus wall but remains constant at the peristaltic wall for increasing values of ${\sigma }_{l}$.
- The sinusoidally advancing wave observed in the graphs of wall shear stress consists of different amplitude crest and trough. The existence of multi-thrombosis in this tube is the reason for this distinct amplitude of crest and trough.
- The crest with greater amplitudes depict the position of multi-thrombosis while the once with low amplitude reveal the location having no thrombus.
- A clear picture of a sinusoidal wave is seen at one end and multi-thrombosis at another end in the streamlines graph.

## List of symbols

$\left(\overline{R},\overline{Z}\right)$: Cylindrical-coordinate system
$\left(\overline{U},\overline{W}\right)$: Velocities along the radial and axial coordinate
$a{R}_{0}$: Radius of catheter
${R}_{0}$: Exterior tube’s radius
$b$: The amplitude of the sinusoidal wave
$${\lambda }_{l}$$: Wavelength $$(l=1,2,3)$$
$\dot{\gamma }$: Rate of shear
${\lambda }_{2}$: Time retardation parameter
$${B}_{r}$$: Brinkman number
$c$: Wave characteristic Speed
${\upsigma }_{l}$: Maximum thrombus heights $(l=1,2,3)$
${z}_{{d}_{l}}$: Thrombus axial translation $(l=1,2,3)$
ϕ: Amplitude to mean radius ratio
${d}_{l}$: Position of clot’s $(l=1,2,3)$
${\lambda }_{1}$: Relaxation to retardation times ratio
$a$: Inner tube to outer tube Radius ratio $(0<a<1)$
